# Omental Lymph Node Transfer for Lymphedema Patients: A Systematic Review

**DOI:** 10.7759/cureus.6227

**Published:** 2019-11-25

**Authors:** Antonio J Forte, Gabriela Cinotto, Daniel Boczar, Maria T Huayllani, Sarah A McLaughlin

**Affiliations:** 1 Plastic Surgery, Mayo Clinic Florida - Robert D. and Patricia E. Kern Center for the Science of Health Care Delivery, Jacksonville, USA; 2 Surgery, Mayo Clinic Florida - Robert D. and Patricia E. Kern Center for the Science of Health Care Delivery, Jacksonville, USA

**Keywords:** volt, lymph node transfer

## Abstract

Lymph node transfer is a surgical treatment that is becoming more prevalent. The lymph nodes from the groin and neck are most frequently used. Iatrogenic lymphedema can be a consequence of the dissection of the groin nodes; thus, some surgeons prefer to use the neck as a donor site. Literature reporting surgical algorithms for the treatment of lymphedema is scarce. Thus, we conducted a systematic review of vascularized omentum lymph node transfer (VOLT) in patients with lymphedema to provide more information about this increasingly common procedure. We hypothesize that the analyzed studies will show that VOLT has positive outcomes. Two reviewers (G.J.C., D.B.) performed independent searches using the PubMed database without timeframe limitations initially through title and abstract descriptions and then by full-text review. The search was done using the following keywords: Breast cancer lymphedema OR lymphedema AND lymph node transfer OR lymph node flap OR lymph node graft AND omental OR omentum OR gastroepiploic. Eligibility criteria included publications evaluating patients with lymphedema in the upper extremity and lower extremity, who underwent VOLT. Our search yielded 35 potential papers in the literature, but only six studies fulfilled the study eligibility criteria. The total number of patients was 137. Three studies described single VOLT, two studies described double VOLT and one study described two cohort patients, one that was treated with single VOLT and another one that was treated with double VOLT. Postoperative reduction of arm circumference, arm volume, and symptoms of the upper extremity were reported in all patients. Nonetheless, in one study, seven patients did not notice any extremity circumference reduction during the follow-up period and four patients noticed an increase in arm volume. Flap loss was reported by two authors in a total of two patients. Overall, patients experienced successful lymphedema treatment with VOLT. All authors presented results with reduced circumferential size of the affected upper and lower limbs, as well as reduction of the infectious intercurrences, such as cellulitis, with a small incidence of associated complications.

## Introduction and background

Lymphedema is a chronic and progressive disease caused by the impairment of the lymphatic system with the accumulation of proteins in the interstitial fluid, adipose tissue hypertrophy, and fibrosis. Lymphedema is classified as primary (congenital or idiopathic) or secondary [1-2]. Early diagnosis and treatment reduces morbidity and mortality and may help prevent irreversible chronic changes in the limb [3]. Identifying risk factors aids in the prevention of disease onset.

Physiologic and excisional procedures to treat lymphedema refractory to conservative therapy have been described [4-5]. The physiologic procedures are lymphovenous anastomosis and lymph node transfer (LNT). The excisional procedures are the radical reduction and preservation of perforators (RRPPs) and suction-assisted lipectomy (SAL).

Lymph node transfer is a surgical treatment that is gaining more popularity [6-7]. The lymph nodes from the groin and neck are most frequently used. Iatrogenic lymphedema can be a consequence of the dissection of the groin nodes; thus, some surgeons prefer to use the neck as a donor site [8]. The vascularized omentum lymph node transfer (VOLT) flap is another resource that provides lymph nodes for lymphedema treatment. Because of the immunogenic and angiogenic properties, the omentum is a better choice for patients with lymphedema associated with cellulitis [9]. The intra-abdominal donor site is also a good option for patients who have limited donor sites or have not responded to other treatments [10]. The vascular endothelial growth factor C, produced by the omental lymph nodes flap, promotes lymphangiogenesis inducing the recanalization of the lymphatic vessels inside the recipient set and the LNT transferred [5,11-13].

Literature reporting surgical algorithms for the treatment of lymphedema is scarce. Thus, we conducted a systematic review of VOLT in patients with lymphedema to provide more information about this increasingly common procedure. We hypothesize that the analyzed studies will show that VOLT has positive outcomes.

## Review

Materials and methods

Two reviewers (G.C., D.B.) performed independent searches using the PubMed database without timeframe limitations. Initially, through title and abstract descriptions and then by a full-text review. Disagreements regarding article identification and final selection for study inclusion were resolved by another reviewer (A.J.F.). The search was done using the following keywords: breast cancer lymphedema OR lymphedema AND lymph node transfer OR lymph node flap OR lymph node graft AND omental OR omentum OR gastroepiploic. The bibliographic reference list of the studies that fulfilled the study eligibility criteria was also examined in order to include articles not present in our initial search. This study followed the guidelines outlined in the Preferred Reporting Items for Systematic reviews and Meta-Analyses (PRISMA flowchart).

Selection criteria

Eligibility criteria included publications evaluating patients with lymphedema in the upper extremity and lower extremity, who underwent VOLT, including all subtypes. Therefore, we excluded papers that did not report VOLT as a lymphedema treatment. Abstracts, presentations, reviews, meta-analyses, case reports, nonclinical studies, and studies without descriptive outcomes were also excluded.

Data extraction and processing

Extracted data included the year of study, country, population, intervention, circumference/volume-reduction/symptoms, lymphoscintigraphy, and complications. Data extraction from articles, tables, and figures was performed by two reviewers (G.C., D.B.), with the accuracy of data entry confirmed by an additional reviewer (A.J.F.).

Results

**Figure 1:**
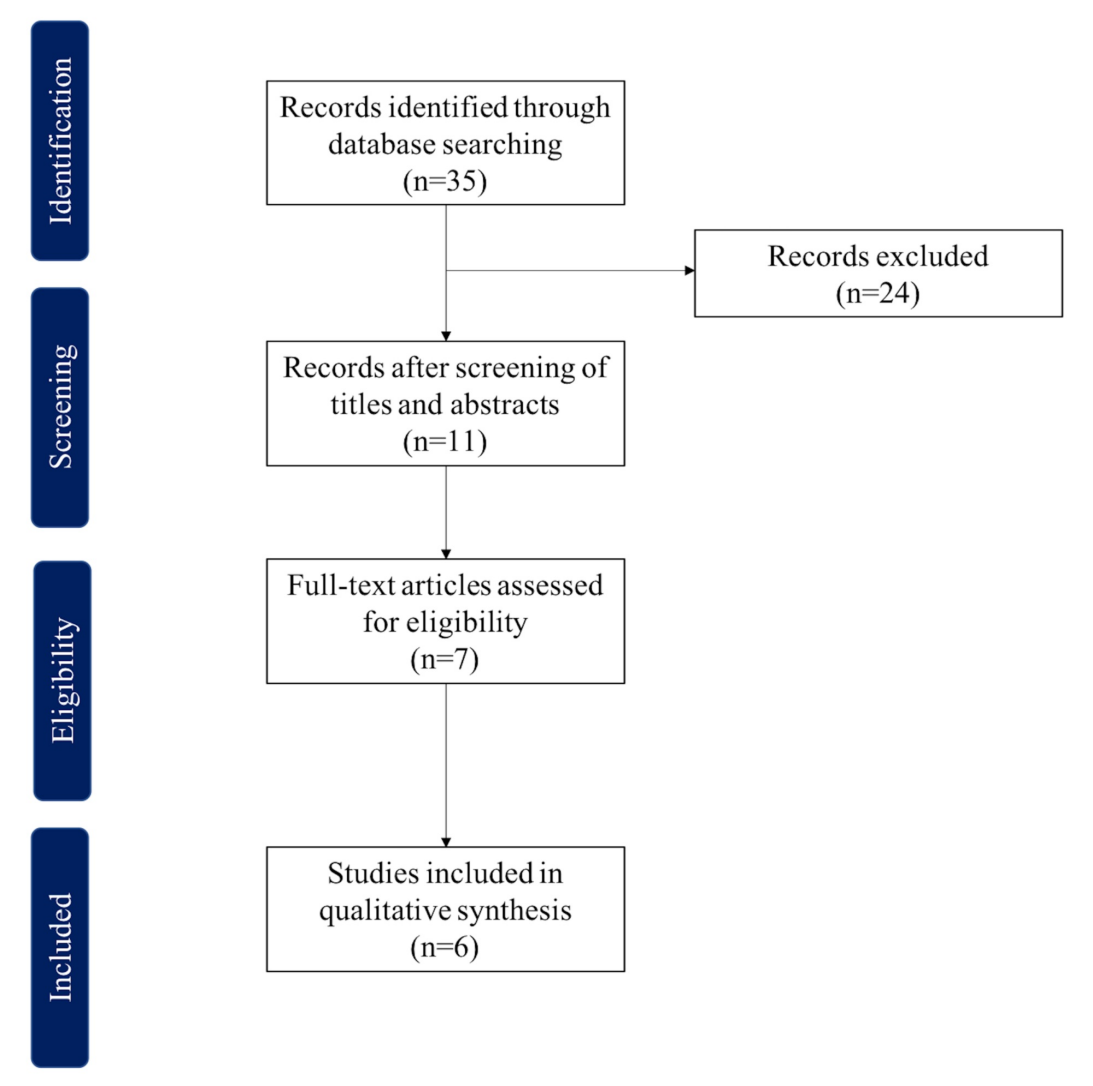
PRISMA flowchart PRISMA: Preferred Reporting Items for Systematic Reviews and Meta-Analyses

The search yielded 35 potential papers (Figure 1) but only six studies fulfilled the study eligibility criteria (Table 1) [4,6-7,14-16]. All included studies were published from 2017 to 2019, in various countries: two studies from China, two studies from the United States, one from Peru, and one from Iran. The total number of patients was 137. The patients’ ages ranged from 18 to 73 years and the follow-up period ranged from 0.5 to 48 months. The population included 88 patients with upper extremity lymphedema, 78 of which had lymphedema after breast cancer treatment, 48 patients had lower extremity lymphedema, and two patients had breast lymphedema.

**Table 1:** Studies Analyzing the Use of Lymph Node Transfer in Lymphedema Treatment VLNT: Vascularized Lymph Node Transfer, Retrospective; VOLT: Vascularized Omental Nymph Node Transfer; RRPP: Radical Reduction with Preservation of Perforators; BRC: Breast Cancer Related; ISL, International Society of Lymphology

Author	Demographic Data	Follow- up Period Mean (Range)	Age. Mean (Range)	Population	Intervention	Circumference/Symptomatic/Volume Reduction	Lymphoscintigraphy	Complications
Nguyen et al. [14] 2017 USA	42 patients *Females: 37 *Males: 5	14 (3–32) months	*52 (18–73) years old	*BRC: 16 *Gynecologic cancer: 12 *others:14 *Upper extremities: 19 *Lower extremities: 24	*Vascularized Free Omental Lymphatic Flap	*Improvements in swelling, fatigue, heaviness, tightness, stiffness, sleep loss, aching *Volume reduction: 74%	*Performed preoperatively and the flap inset postoperatively	*Flap loss: 1/Not reporting improvement: 7 patients/Patients increasing in volume: 4/Arteriovenous fistula creation of the left gastroepiploic vessels: Nine patients (21%) *Donor site complications: Pancreatitis: 1patient/Nasogastric tube replacement for an additional 48 hr: 1 patient * Recipient site: Hematomas: 2/Seromas: 2
Ciudad et al. [4] 2017 China	10 Female patients	14.7 (range 9–19) months	54.8 years (range 48–62 years)	*BRC: 5 patients *Gynecological cancer-related: 5 patients ISL: *Stage II: 2 patients *Stage III: 8 patients	Laparoscopically harvested right gastroepiploic lymph node flap	*Mean circumference reduction rate: 39.5% +- 1.8% (range, 35.5–42.3%) *Improvement of the functional, appearance, symptoms and mood scores	*Perioperative lymphoscintigraphy: transferred lymph node viability and lymphatic transport improvement	*Partial skin graft loss requiring regrafting at postoperative: 1 patient
Ciudad et al. [7] 2017 China	*7 patients	9.7 months (range, 8–11 months)	*53.1 years (range, 42–65 years)	*BRC: 4 patients *Lower limb pelvic cancer‐related lymphedema: 3 patients	* Double gastroepiploic vascularized lymph node transfer	*Mean circumference reduction rate was 43.7 ± 2.5% along the entire limb (P	NS	*No donor or recipient site complication
Kenworthy et al. [15] 2018 USA	*38 patients	9.6 (0.5-24) months	*54.9 (27-72) year-old	*BRC: 62.5% *Gynecologic cancer-related: (12.5%) *Others: 25%	*Vascularized Omentum Lymph node Transfer (VOLT)	*Cellulitis reduction: 13.2% *Observed clinical improvement of lymphedema	Physiologic function: 50% of patients on lymphoscintigraphy *Physiologic function on ICG lymphangiography: 20%	*Single VOLT: *Donor site complications: Transient pancreatitis 1 patient (2.6%)/Ileus: 2 patients for 4 to 5 days (5.3%)/Nasogastric tube for 24 hrs: 1 patient *Recipient site complications: Flap loss: 1(1.9%)/Hematoma: 2 (3.7%)/Avulsion of the arterial anastomosis: 1 patients *Double VOLT: *Recipient Site: Hematoma: 1 (3.1%)
Ciudad et al. [6] 2019 Peru	16 patients *15 Female *1 male	14.2 months (range, 12‐19)	* 58.75 ± 9.8 years (range, 36‐67)	*Primary lymphedema: 1 *BRC: 6 *lower extremity lymphedema ovarian or cervical cancer related:10	Gastroepiploic VLNT+ two levels of inset and modified RRPP	*Mean circumference reduction was 70.8% ± 5.9% (range, 62%‐84%) *Improvement in function, appearance, symptoms, and mood	Post-operative Lymphoscintigraphy: significant improvement in the lymphatic drainage	*Recipient‐site: Paresthesia:3/Hyperesthesia: 1/Seroma: 1
Mousavi et al. [16] 2019 Iran	24 patients	1 to 4 years	* 48.7 years (range, 35‐70 years)	*BRC: 100%	*Vascularized lymph nodes were transferred from the gastroepiploic lymph nodes to the volar forearm	*Cellulitis was significantly reduced *Circumferential size of the upper extremity was significantly reduced *Significant improvement, satisfaction, function, and acceptable appearance	NS	No flap failures / Flap required early re‐exploration due to evidence of venous compromise: 1

Three studies described single VOLT [4,14,16], two studies described double VOLT [6-7], and one study described two cohort patients, one that was treated with single VOLT and another one that was treated with double VOLT [15]. The recipient site was variable according to the lymphedema area. Postoperative reduction of arm circumference, arm volume, and symptoms of the upper extremity were reported in all patients. Nonetheless, in one study, seven patients did not notice any extremity circumference reduction during the follow-up period, and four patients noticed an increase in arm volume [14]. Flap loss was reported by two authors in a total of two patients [14-15]. Nguyen et al. reported venous hypertension [14], and Kenworthy et al. reported a soft-tissue infection after a single VOLT procedure as a cause of flap failure [15]. Moreover, arteriovenous fistula [14], seroma [6,14], hematoma [14-15], nasogastric tube for additional 24-48 hours [14-15], pancreatitis [14-15], partial skin graft loss [4], paresthesia [6], and hyperesthesia were reported as complications by five authors [6]. Lymphoscintigraphy was described as a diagnostic method used during the follow-up period to evaluate the patients by five authors [4,6-7,14-15]. The quality of life scores were also evaluated in some of the studies [4,6-7].

Discussion

The literature described different lymph node donor sites: the supraclavicular fossa, groin, intra-abdominal, submandibular region, lateral thoracic area, and axilla donor sites [4,17]. There is no evidence indicating one site’s superiority over the others. The first authors that described the lymphedema treatment in animals with intra-abdominal-omental tissue were Goldsmith and De Los Santos [18] while the omental transposition to the axilla was reported for the first time by Nakajima et al. [19].

In this systematic literature review, we found that lymphedema improved considerably after VOLT, which suggests it as a promising technique. The understanding of lymphedema and the evolution of laparoscopic and microsurgery techniques have made VOLT a safe alternative to treat lymphedema, mainly because some patients had refractory lymphedema, did not have a successful lymph node transfer, or had limited donor sites [10]. The objective is to use the lymph nodes from donor sites that have a larger number of lymph nodes with multiple drainage sources or use flaps that only remove the lymph nodes that are not crucial to the drainage functions of the donor region. The plentitude of lymph nodes at the omentum and the morphology of the blood vessels make this flap amenable to division [6]. The disadvantages of VOLT include the risk of incisional hernia, hypertrophic scars, adhesions, bowel obstruction, and pancreatitis, which is a rare complication associated with VOLT. The gastroepiploic vessels are thinner compared to vascularized lymph node Transfer (VLNT) from other sites; therefore, they are more prone to kinking. The flap could be performed by laparoscopy or by open laparotomy [6,16].

From this literature review, we noticed differences between the techniques that were used to perform VOLT. In one direction, the authors performed single VOLT, which showed good results and with a reduced surgical time [4,14-16]. In the other direction, the authors performed double VOLT, which is a double-level inset from a single lymph node flap transfer (LNFT) [6-7,15]. The advantage of this procedure is the opportunity to split the flap into two levels, avoiding the possible morbidity associated with a second harvest site. Moreover, this allows the surgeon to chose two different recipient sites.

The choice of the recipient site in lymph node transfer is controversial [20]. Patients with previous inguinal or axillary lymph node dissection can be favored with the re-establishment of the lymphatic network with an orthotopic VLNT [6]. Moreover, it allows for scar release or removal through the same incision. Decompression of the axillary vein can also be achieved and these patients will not require a skin flap. However, the stagnation of fluid is more prominent at the distal extremity and increases the risks of degenerative lesions caused by lymphedema, which is why some surgeons prefer distal placement. According to Ciudad et al., the placement of the second LNT at the center of the extremity combined with a distal lymph node transfer (LNT) improves the pump-absorbing lymphatic fluid in two sites and gives the extremity a double site for lymphangiogenesis. The midlevel inset site, such as the proximal recipient site, avoids the need for a skin graft [6-7].

Ciudad et al. published in 2019 the first study that associated double VOLT with RRPPs in patients with advanced lymphedema [6]. One flap was placed at the midlevel at the cubital fossa and the other inset was placed at the wrist where the RRPPs cannot be performed improving the lymphatic drainage of the extremity. The preservation of the superficial vein system and the scar release undertaken in patients with secondary lymphedema are the major modifications that the author showed on the review. The mean circumference reduction was 70.8%±5.9% (range, 62%-84%), being the higher reduction between the reviews from this author [6]. Ciudad et al. performed two more studies: one with a single VOLT and the other with double VOLT [4,7].

The patients with dense fibrosis and excess adipocutaneous tissue benefit from the additional treatment with an excisional procedure such as RRPPs and SAL [6]. The SAL procedure is preferred over the RRPPs; however, the compression garments made the SAL a selective method that requires a periodic follow-up to achieve good results. The advantages of the RRPPs as compared to SAL are the removal of fibrotic tissue, the reduction of redundant skin, and the preservation of perforators, which is essential to maintain the nutrition of the skin [6].

Venous hypertension at the flap is commonly associated with conventional microanastomoses. To avoid it, Kenworthy et al. suggested anastomosis of the distal gastroepiploic vein to a second recipient vein [15]. The symptom is clinically evident, with pulsation at the end of the distal gastroepiploic vein [15].

Our systematic review reports PubMed-based manuscripts to date that evaluated VOLT procedures in the English-language literature. We recognize the presence of several limitations to our study. First, the small number of studies and, consequently, a small cohort. Second, the lack of prospective randomized studies and the nonstandardization of the obtained results make it difficult to establish protocols. Last, the absence of objective measurement of arm circumference and volume, as well as cellulitis rate reduction, impeded a quantitative evaluation of outcomes. In addition, the follow-up duration in the studies is too little to evaluate the persistent benefit of these procedures. However, despite these limitations, we believe that VOLT is a promising technique with good results. We suggest future retrospective and prospective studies enrich the evidence to support this practice.

## Conclusions

Overall, patients experienced successful lymphedema treatment with VOLT during the follow-up period (ranging from 0.5 to 48 months). All authors present the results with the reduced circumferential size of the affected upper and lower limbs, as well as the reduction of infectious intercurrences, such as cellulitis, with a small incidence of associated complications.
